# Association of the C-Reactive Protein–Triglyceride–Glucose Index with Stroke–Heart Syndrome and Clinical Prognosis in Patients Undergoing Endovascular Treatment

**DOI:** 10.3390/jcdd13050179

**Published:** 2026-04-25

**Authors:** Wenjie Chen, Xuesong Bai, Tao Wang, Liqun Jiao, Liyong Zhang, Hong Li

**Affiliations:** 1 Department of Cardiology, Beijing Chao-Yang Hospital, Capital Medical University, Beijing 100020, China; wenjiechen@mail.ccmu.edu.cn; 2 Department of Neurosurgery, Beijing Xuan-Wu Hospital, Capital Medical University, Beijing 100053, China; 3 Department of Neurosurgery, Liaocheng People’s Hospital, Shandong First Medical University, Liaocheng 252000, China

**Keywords:** stroke–heart syndrome, C-reactive protein–triglyceride–glucose index, acute ischemic stroke, endovascular thrombectomy

## Abstract

Background: Stroke–heart syndrome (SHS), particularly acute myocardial injury, is a critical complication following acute ischemic stroke (AIS). The C-reactive protein–triglyceride–glucose index (CTI) integrates inflammatory and metabolic parameters but remains unexplored in the context of post-stroke cardiac complications. This study investigated whether CTI predicts cardiac injury patterns and 90-day clinical outcomes in AIS patients. Methods: A two-center retrospective cohort study was conducted in AIS patients undergoing endovascular treatment (EVT). Cardiac troponin I (cTnI) trajectories were classified into: no injury, non-dynamic elevation, and dynamic elevation. The primary endpoint was poor functional status at 90 days (modified Rankin Scale [mRS] 3–6); the secondary endpoint was 90-day all-cause death. Results: Among 493 individuals (median age 69 years, 42% female), higher baseline CTI was associated with a greater likelihood of dynamic troponin elevation (adjusted odds ratio [aOR] per 1-unit increase = 1.56 (1.26–1.94); *p* < 0.001). Patients with dynamic elevation had significantly worse outcomes compared to those with no injury. Elevated CTI was an independent predictor of 90-day poor functional outcome (Q4: aOR = 3.04 (1.53–6.05); *p* < 0.001) and mortality (Q4: aOR = 2.82 (1.33–6.00); *p* = 0.007). Conclusions: In EVT-treated AIS patients, the CTI is a predictor of SHS and adverse 90-day outcomes. This easily calculable index may help identify individuals at higher risk of cardiac complications after AIS.

## 1. Introduction

Acute ischemic stroke (AIS) commonly precipitates a range of cardiac manifestations—encompassing arrhythmias, myocardial injury, and ventricular dysfunction—collectively designated as Stroke–heart syndrome (SHS). This syndrome substantially influences post-stroke prognosis and represents a major contributor to mortality in AIS populations [1,2]. Within the spectrum of SHS, acute myocardial injury exhibits high prevalence and correlates with adverse clinical trajectories. While elevated cardiac troponin I (cTnI) is the biochemical hallmark of this injury, interpreting cTnI levels in stroke patients is challenging [3,4]. A single elevated value may reflect chronic comorbidities (e.g., stable coronary artery disease), whereas dynamic changes in cTnI levels are more indicative of the acute, stroke-induced cardiac damage characteristic of SHS [5]. Therefore, distinguishing between chronic stable elevation and the acute dynamic trajectory is crucial for identifying the SHS.

The pathogenesis of SHS is complex and related to the “brain-heart axis”. It is believed that mechanisms such as a surge in adrenaline, autonomic nerve dysfunction, and oxidative stress can trigger neurogenic myocardial damage [6,7]. Recent research indicates that systemic inflammatory metabolic imbalance may play a crucial role in SHS. Under the conditions of AIS, the interaction between systemic inflammation and insulin resistance will exacerbate endothelial dysfunction and myocardial fragility [8,9]. The C-reactive protein (CRP)–triglyceride (TG)–glucose index (CTI) has been proposed as a new composite marker that captures both inflammatory and metabolic status [10,11]. Although higher CTI has been linked to long-term cardiovascular risks, its role in the acute phase of stroke—specifically in the context of EVT-treated patients—remains unclear [12]. Furthermore, whether CTI can predict the SHS has not been explored.

Therefore, we conducted a two-center retrospective cohort study of patients with anterior-circulation large-vessel occlusion (LVO) undergoing EVT. Our objectives were to examine the relationship between CTI and: (1) the specific phenotypes of SHS, characterized by dynamic cTnI trajectories (based on paired cTnI measurements at admission and 72 h), and (2) clinical endpoints, specifically 90-day functional disability and 90-day all-cause death.

## 2. Materials and Methods

### 2.1. Study Design and Population

In this two-center retrospective analysis, we evaluated consecutive AIS patients with anterior-circulation LVO who received EVT between June 2020 and June 2023 at comprehensive stroke facilities. Patients were eligible for inclusion if they met the following criteria: (1) age ≥ 18 years; (2) AIS confirmed by neuroimaging; (3) occlusion of the intracranial internal carotid artery (ICA) or middle cerebral artery (MCA, M1/M2 segments) confirmed by CT angiography, MR angiography, or digital subtraction angiography (DSA); (4) EVT performed within guideline-recommended time windows (within 6 h of symptom onset, or 6–24 h based on advanced imaging selection) [13]; (5) cTnI measurements obtained at admission and repeated at 72 h; and (6) completion of 90-day follow-up.

**Figure 1:**
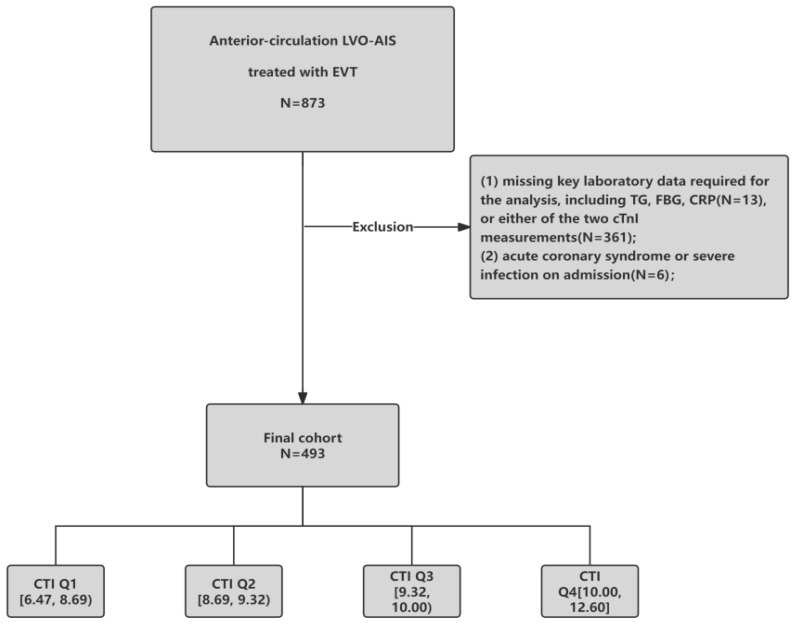
Flowchart of this study.

Exclusion criteria were: (1) missing key laboratory data required for CTI calculation (CRP, TG, or fasting blood glucose [FBG]); (2) evidence of primary acute coronary syndrome or chest pain preceding stroke onset; or (3) active severe infection at admission. The study protocol was approved by the Institutional Review Boards of both participating centers. Due to the retrospective nature of the study, the requirement for written informed consent was waived. The patient selection process is detailed in the study flowchart (Figure 1).

### 2.2. Clinical and Procedural Data Collection

Baseline demographic and clinical characteristics were extracted from maintained stroke registries, supplemented by electronic health record review. Variables included age, sex, vascular risk factors (coronary artery disease (CAD), hypertension, hyperlipidemia, diabetes mellitus, atrial fibrillation (AF)), prior cerebrovascular events, and smoking history. Initial stroke severity was quantified using the National Institutes of Health Stroke Scale (NIHSS). Baseline infarct burden was evaluated via the Alberta Stroke Program Early CT Score (ASPECTS) on non-contrast imaging [14].

Stroke mechanism was classified per the Trial of ORG 10172 in Acute Stroke Treatment (TOAST) criteria into cardioembolism, large-artery atherosclerosis, or other/undetermined etiologies [15,16]. Vessel occlusion location was categorized as internal carotid artery (ICA), M1, or M2 segment [17].

Procedural parameters comprised initial thrombectomy technique (no device pass, aspiration, stent retriever, or combined strategy) and final reperfusion status graded by modified Thrombolysis in Cerebral Infarction (mTICI) scale [18]. Successful reperfusion was defined as mTICI grade 2b–3 [19,20].

Post-procedural complications encompassed symptomatic intracranial hemorrhage (sICH) and malignant cerebral edema (MCE) [21]. sICH was identified per Heidelberg Bleeding Classification criteria [22,23,24]. MCE was operationalized as: (1) hypodense parenchymal involvement affecting ≥50% of middle cerebral artery territory and/or regional swelling/herniation; and (2) midline shift ≥5 mm at the septum pellucidum or pineal gland with basal cistern effacement [25].

### 2.3. Laboratory Measurements and Variable Definitions

#### 2.3.1. Blood Sample Collection and Timing

Blood samples were collected at three standardized timepoints:

(1) Admission samples (pre-EVT): CRP and the first cTnI measurement were obtained from venous blood samples drawn at the time of emergency presentation, prior to the EVT procedure. The median time from symptom onset to blood sampling was 3.2 h (interquartile range [IQR]: 1.7–5.2 h).
(2) Fasting samples (post-admission): TG and FBG were measured from venous blood samples collected on the morning following admission, after an overnight fast of at least 8 h. Fasting blood samples were obtained at a median of 17 h (IQR 13–23 h) post-admission. All fasting samples were collected after EVT completion.
(3) 72 h follow-up samples: The second cTnI measurement was obtained at 72 h after admission to assess dynamic changes in cardiac injury markers.

This staggered sampling protocol was designed to balance clinical feasibility with measurement accuracy (Table S1). CRP is measured at admission to obtain the baseline status. Fasting samples need to be prepared overnight, so they are collected in the early morning of the second day after admission.

#### 2.3.2. Definition and Grouping of CTI

The CTI is used to reflect the comprehensive level of an individual’s inflammatory and metabolic status. The calculation formula is as follows: CTI = 0.412 × Ln(CRP (mg/L)) + Ln [TG (mg/dL) × FBG (mg/dL)/2] [10].

For analysis, CTI was treated both as a continuous variable and categorized into quartiles (Q1–Q4) based on the distribution within the study cohort.

#### 2.3.3. cTnI Testing and Trajectory

cTnI was measured using a high-sensitivity assay at two timepoints: at admission (pre-EVT) and at 72 h after admission. Both participating centers used the same high-sensitivity assay platform. The institutional upper reference limit (URL, 99th percentile) was 0.034 ng/mL. To characterize the phenotype of SHS, troponin trajectories were classified into three ordinal categories based on the dynamic change between the two timepoints:

(1) No myocardial injury: Both cTnI measurements ≤ URL.
(2) Non-dynamic elevation: At least one cTnI measurement > URL, without evidence of acute progression. This included patients whose 72 h cTnI decreased by ≥20% relative to admission (suggesting resolving injury) or whose values changed by <20% in either direction (suggesting chronic stable change).
(3) Dynamic elevation: At least one cTnI measurement > URL with evidence of acute progression, defined as a ≥20% relative increase from admission to 72 h.

The 20% relative change threshold for defining dynamic patterns was based on the Fourth Universal Definition of Myocardial Infarction recommendations for distinguishing acute from chronic injury [26]. In regression analyses, troponin trajectory was modeled as an ordinal outcome reflecting increasing severity of cardiac vulnerability.

### 2.4. Outcomes

The primary clinical endpoint was unfavorable functional status at 90 days, operationalized as modified Rankin Scale (mRS) scores of 3–6. mRS assessments were performed by trained stroke neurologists and stroke research nurses. For patients who cannot come to our center for follow-up, we obtain the mRS from the cooperating community hospitals or conduct follow-up by phone. The assessment follows standardized criteria: 0: No symptoms; 1: No significant disability despite symptoms; able to perform all usual duties; 2: Slight disability; unable to perform all previous activities but able to look after own affairs without assistance; 3: Moderate disability; requires some help but able to walk without assistance; 4: Moderately severe disability; unable to walk without assistance and unable to attend to bodily needs without assistance; 5: Severe disability; bedridden, incontinent, requires constant nursing care; 6: Death.

The secondary outcome was 90-day all-cause death.

### 2.5. Statistical Analysis

Continuous data were examined for normality via the Shapiro–Wilk test. Normally distributed variables are expressed as mean ± standard deviation (SD), while non-normally distributed variables are reported as median with interquartile range (IQR). Categorical data are summarized as frequencies and percentages. Between-quartile comparisons employed the Kruskal–Wallis test for continuous measures and Pearson’s χ^2^ test for categorical measures.

We applied ordinal logistic regression to assess the relationship between CTI and troponin trajectory patterns, with results presented as odds ratios (ORs) and 95% confidence intervals (CIs). For the primary endpoint (90-day mRS 3–6) and secondary endpoint (90-day mortality), binary logistic regression evaluated associations of CTI and cTnI trajectory with outcomes. CTI was analyzed both continuously and categorically (by quartiles); linear trends across quartiles were examined using *p* for trend.

Three sequential adjustment models were constructed:

- Model 1: Crude analysis without covariate adjustment
- Model 2: Adjusted for patient demographics and vascular risk factors (age, sex, hypertension, diabetes mellitus, hyperlipidemia, CAD, AF, prior stroke, and smoking status)
- Model 3: Additionally adjusted for stroke-specific characteristics and procedural factors beyond Model 2 covariates—including occlusion site, TOAST classification, baseline NIHSS score, baseline ASPECTS, initial thrombectomy approach, final reperfusion grade (mTICI 2b–3 vs. 0–2a), sICH, and MCE

To assess whether CTI has incremental predictive value, we compared the discriminatory performance of each model through ROC curve analysis. Variables included in the basic model: the same as Model 3; Model 4: CRP + basic model; Model 5: CTI+ basic model.

To assess whether the prognostic value of CTI operates through subsequent cardiac injury (indirect pathway) or through an independent mechanism (direct pathway), we conducted a causal mediation analysis. The mediator variable was the dynamic increase in troponin, and for the convenience of analysis, the trajectory was classified as a binary variable. Specifically, patients were divided into those with dynamic increase and those without dynamic increase (including no myocardial injury and non-dynamic elevation patterns). All covariates in Model 3 were adjusted. A bootstrap resampling of 1000 times was used.

Subgroup analyses were conducted to assess associations of CTI (continuous) with 90-day function outcome and mortality; interaction terms were used to test for effect modification (*p* for interaction). Sensitivity analysis was conducted by stratifying based on the cTnI trajectory to verify the robustness of the association between CTI and outcomes. Additionally, stratification was performed according to estimated glomerular filtration rate (eGFR) to explore the impact of CTI on outcomes under different renal function states.

Given the retrospective nature of the study, no formal a priori sample size calculation was performed; the sample size was determined by the availability of eligible consecutive patients during the study period. Regarding data quality, patients with missing key variables for the primary exposure (CTI) or outcome assessment were excluded as per the study design. Among the final analytic cohort, there were no missing data for other covariates, and 90-day functional status was ascertained for all patients (0% loss to follow-up). All statistical analyses were conducted with R software (version 4.3.2). Two-tailed tests were performed, with significance defined as *p* < 0.05.

## 3. Results

### 3.1. Baseline

Overall, 873 patients underwent screening. We excluded 361 patients without paired cTnI measurements (admission and 72 h), 13 patients with missing TG/FBG/CRP data, and 6 patients with infection or acute coronary syndrome at admission. The final analytic cohort included 493 patients.

Patients were categorized by CTI quartiles: Q1 [6.47, 8.69) (*n* = 123), Q2 [8.69, 9.32) (*n* = 123), Q3 [9.32, 10.00) (*n* = 123), and Q4 [10.00, 12.60] (*n* = 124). Age, baseline NIHSS, and baseline ASPECTS did not differ significantly across CTI quartiles (all *p* > 0.05). Sex distribution differed across quartiles (*p* = 0.012), and the prevalence of diabetes mellitus and hyperlipidemia increased with higher CTI (both *p* < 0.001). TOAST subtype distributions differed across quartiles (*p* = 0.001). No significant between-quartile differences were observed for hypertension, CAD, AF, smoking status, prior stroke, occlusion site, mTICI grade, sICH, or post-EVT MCE (all *p* > 0.05). Troponin trajectory differed significantly across CTI quartiles (*p* < 0.001), and the proportion of patients with dynamic elevation increased with higher CTI (Table 1).

### 3.2. Association Between CTI and cTnI Trajectory

**Figure 2:**
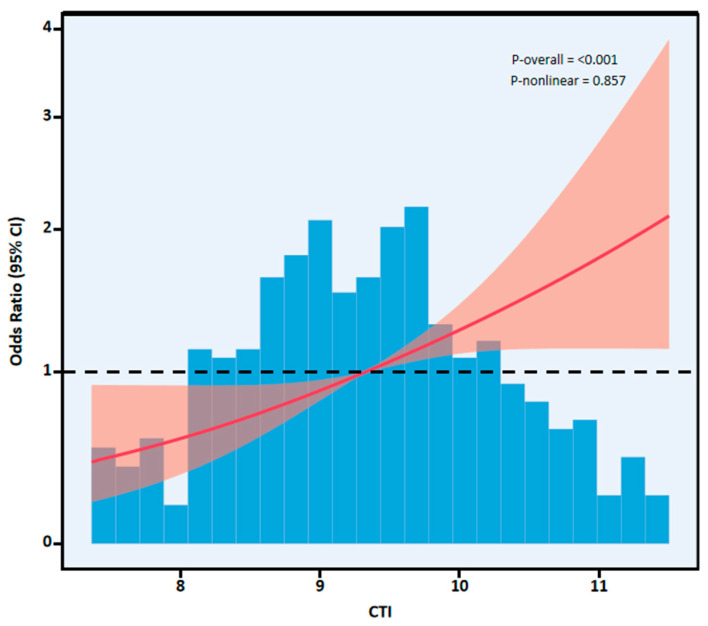
Restricted cubic spline (RCS) analysis of the association between CTI and troponin trajectory. Red represents the confidence interval.

Ordinal logistic regression showed a positive relationship between CTI and more severe cTnI trajectory categories (Table 2). Treating CTI as continuous, each 1-unit increase in CTI was associated with higher odds of being in a worse cTnI trajectory category in the fully adjusted analysis (Model 3: OR 1.56, 95% CI 1.26–1.94; *p* < 0.001). In quartile-based analysis (reference: Q1), higher CTI quartiles were associated with worse cTnI trajectory in a dose–response manner; in Model 3, Q4 remained significant (Q4: OR 3.49, 95% CI 1.87–6.51; *p* < 0.001), with a significant trend across quartiles (*p* for trend < 0.001). The restricted cubic spline (RCS) showed a significant, approximately linear association between CTI and the cTnI trajectory (*p*-overall < 0.001; *p*-nonlinear = 0.857) (Figure 2).

### 3.3. Functional Outcome and Mortality Across CTI Quartiles

**Figure 3:**
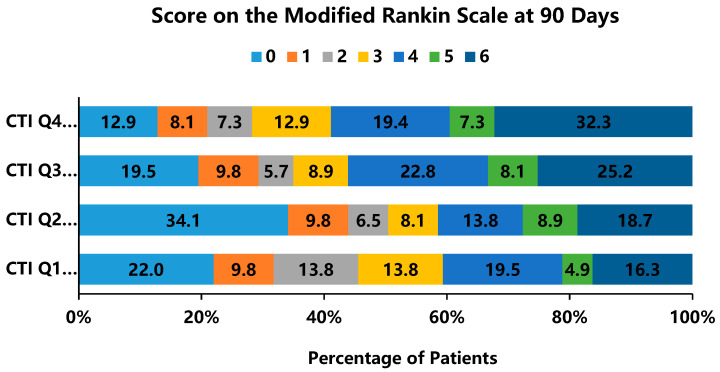
Distribution of modified Rankin scale (mRS) scores at 90 days stratified by CTI quartiles.

Clinical outcomes differed significantly across CTI quartiles (Table S2). Poor functional status (mRS 3–6) at 90 days increased from 54.5% in Q1 to 71.8% in Q4 (*p* = 0.001). 90-day all-cause mortality also showed a progressive increase with higher CTI levels (Q1–Q4: 16.3%, 18.7%, 25.2%, and 32.3%, respectively; *p* = 0.013). The ordinal distribution of mRS scores (Figure 3) demonstrated progressive deterioration with rising CTI: excellent outcomes (mRS 0–1) declined from 31.8% (Q1) to 21.0% (Q4), while death rates (mRS 6) doubled from 16.3% to 32.3%.

### 3.4. Association Between CTI and Clinical Outcomes

#### 3.4.1. Primary Endpoint: 90-Day Functional Disability (mRS 3–6)

Logistic modeling (Table 3) identified CTI as an independent predictor of unfavorable functional status. When modeled as a continuous variable, CTI remained an independent predictor in the fully adjusted model (Model 3: OR 1.47, 95% CI 1.16–1.86; *p* = 0.001). In quartile analyses, compared to the lowest quartile (Q1), patients in the highest quartile (Q4) had a significantly higher risk of poor functional outcome (Model 3: OR 3.04, 95% CI 1.53–6.05; *p* = 0.001).

#### 3.4.2. Secondary Outcome: 90-Day All-Cause Mortality

**Figure 4:**
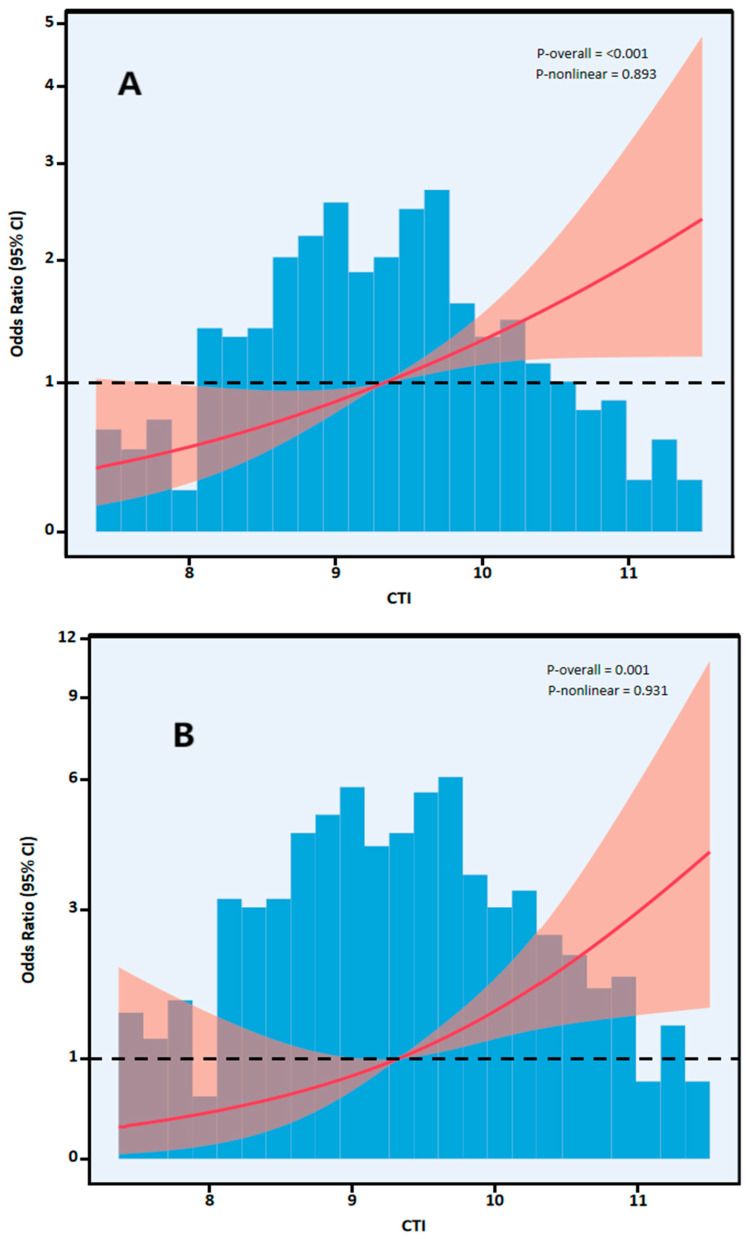
(**A**). RCS analysis of the association between CTI and poor functional outcome; (**B**). RCS analysis of the association between CTI and 90-day mortality. Red represents the confidence interval.

CTI similarly predicted mortality risk. In fully adjusted analysis, continuous CTI was significantly associated with mortality (OR 1.62, 95% CI 1.25–2.11; *p* < 0.001). Patients in Q4 exhibited a nearly 2.8-fold increased risk of death compared to those in Q1 (Model 3: OR 2.82, 95% CI 1.33–6.00; *p* = 0.007), with a significant linear trend across quartiles (*p* for trend = 0.003). RCS analyses verified linear positive associations between continuous CTI and both functional disability (Figure 4A) and mortality (Figure 4B).

#### 3.4.3. Predictive Performance and Incremental Clinical Utility of CTI

**Figure 5:**
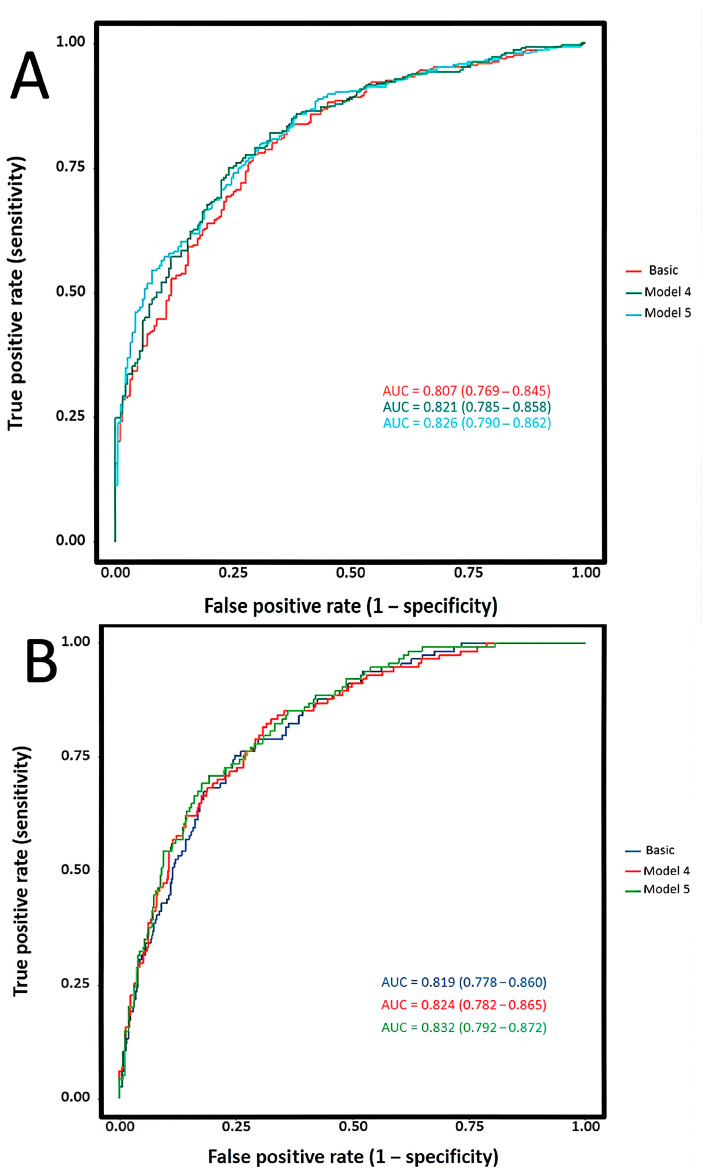
Receiver operating characteristic curves comparing the incremental discriminative value of CTI vs. CRP for clinical outcomes. (**A**) 90-day poor functional outcome (modified Rankin Scale score 3–6). (**B**) 90-day all-cause mortality. The basic model includes covariates from Model 3. Model 4 adds C-reactive protein (CRP) to the basic model. Model 5 adds the CTI to the basic model.

To compare CTI versus CRP, we performed sequential ROC analyses using nested models (Figure 5, Table S3). For 90-day poor functional outcome, the base model yielded AUC 0.807 (0.769–0.845). Adding CRP improved discrimination to 0.821 (*p* = 0.059), while CTI achieved 0.826 (*p* = 0.025). Direct comparison showed CTI’s advantage (ΔAUC = +0.005) was non-significant by DeLong’s test (*p* = 0.497) but significant by likelihood ratio test (*p* = 0.028). For mortality, CRP provided minimal improvement (AUC 0.824, *p* = 0.472), while CTI significantly enhanced discrimination (AUC 0.832, *p* = 0.038). Direct comparison was non-significant (*p* = 0.263), but likelihood ratio testing favored CTI (*p* = 0.015), suggesting CTI captures prognostic information beyond discrimination metrics.

### 3.5. Association Between cTnI Trajectory and Outcomes

cTnI trajectory patterns demonstrated strong prognostic value (Table 4). After comprehensive covariate adjustment, patients exhibiting dynamic cTnI elevation faced markedly elevated risk for both 90-day functional disability (aOR 8.73, 95% CI 4.53–16.83; *p* < 0.001) and mortality (aOR 6.12, 95% CI 3.40–11.19; *p* < 0.001) relative to the no-injury reference group. However, the “non-dynamic elevation” pattern was not significantly associated with worse outcomes compared to the reference group (all *p* > 0.05).

### 3.6. Mediation Analysis

Mediation analysis revealed that CTI’s association with poor functional outcome (total effect: 0.385, 95% CI 0.218–0.562, *p* < 0.001) operated through both indirect and direct pathways (Table 5). The indirect effect mediated through dynamic cTnI elevation accounted for 36.4% of the total effect (ACME = 0.140, *p* < 0.001), while the direct effect independent of troponin dynamics represented 63.6% (ADE = 0.241, *p* = 0.002).

### 3.7. Subgroup Analyses

Subgroup analyses were performed to evaluate the consistency of the association between CTI and clinical outcomes across key baseline characteristics. For 90-day poor functional outcome (Table S4), the association between higher CTI levels and poor outcome remained consistent across all subgroups, including age, sex, and comorbidities. No significant interactions were observed for any of the stratification variables (all *p* for interaction > 0.05). Specifically, the association was not significantly modified by the history of AF (*p* for interaction = 0.992), with similar effect sizes observed in patients with or without AF. Similarly, for mortality (Table S5), the CTI–death association showed consistent directionality and magnitude across subgroups.

### 3.8. Sensitivity Analyses

Given potential confounding by renal dysfunction, we stratified analyses by estimated glomerular filtration rate (eGFR) (Table S6). The CTI-troponin trajectory relationship persisted across renal function categories (*p* for interaction = 0.614).

To assess the robustness of the association between CTI levels and the primary outcome in different troponin trajectories, we also conducted a sensitivity analysis of the 90-day adverse functional outcome (Table S7). The association between higher CTI levels and increased risk of poor functional outcome remained generally consistent across different cardiac injury patterns (*p* for interaction = 0.371). Among patients with no myocardial injury, the highest CTI quartile conferred substantially elevated risk versus the lowest quartile (OR 3.94, 95% CI 1.46–6.71; *p* = 0.018).

## 4. Discussion

In this two-center retrospective analysis of anterior-circulation LVO patients treated with EVT, we investigated relationships between CTI, cardiac injury patterns, and clinical outcomes. Our principal findings demonstrate that elevated CTI associates with dynamic cTnI trajectories—the hallmark of SHS. Moreover, higher CTI independently predicts 90-day functional disability and mortality.

Whether CTI reflects pre-existing chronic dysfunction or acute stress responses is an important consideration. In the hyperacute phase, stress responses can affect all CTI components; therefore, elevated CTI might partially reflect acute stroke severity. However, several findings of this study indicate that the impact of long-term vulnerability is significant: (1) CRP was measured at 3.2 h after symptom onset, when acute-phase elevation is minimal (CRP rises at 4–12 h and peaks at 5–7 days [27]); (2) CTI remained independent after adjustment for multiple severity markers (NIHSS, ASPECTS, reperfusion status, sICH). We acknowledge that the absence of HbA1c or pre-stroke CRP limits definitive distinction between chronic and acute components—a “fundamental challenge for any biomarker measured in the acute phase” [28]. Nevertheless, the convergence of evidence supports CTI’s utility for identifying high-risk patients requiring intensified monitoring.

In addition, the TRELAS study has identified advanced age, structural heart disease, and renal impairment as risk factors for post-stroke troponin elevation [29]. Considering the influence of the above factors on the changes in cTnI after stroke, this study corrected for factors such as CAD and AF. The results showed that CTI remained a powerful predictor of SHS. Additionally, a sensitivity analysis stratified by estimated glomerular filtration rate (eGFR) was conducted (Table S5). We found that the association between CTI and troponin trajectory was consistent across different renal function categories (*p* value for interaction = 0.614), suggesting that the relationship between CTI and cardiac injury in this study may not be mainly driven by renal function impairment.

The association between CTI and SHS observed in our study may be explained by several mechanisms. First, CTI integrates inflammation and endothelial dysfunction. CRP-associated inflammation can aggravate endothelial dysfunction and microvascular injury, potentially increasing myocardial vulnerability to catecholamine surges and hemodynamic fluctuations during severe stroke [30,31]. Second, insulin resistance (reflected by the TyG component of CTI) is linked to impaired myocardial substrate utilization and oxidative stress. In acute illness, this “metabolic inflexibility” may reduce the heart’s tolerance to supply-demand mismatches, promoting injury manifested as dynamic troponin release [32,33]. Third, there is likely a synergistic amplification between inflammation and metabolic dysregulation [34,35].

An important consideration is whether CTI offers advantages over CRP. When added to a comprehensive base model (Figure 5, Table S3), CTI demonstrated significant incremental value for both outcomes (*p* = 0.025 and *p* = 0.038), while CRP showed inconsistent results (*p* = 0.059 and *p* = 0.472). Although direct AUC comparisons were non-significant, likelihood ratio testing favored CTI (*p* = 0.028 and *p* = 0.015). This indicates that the metabolic component provides complementary prognostic information to inflammation. This aligns with the emerging concept of “Cardiovascular-Kidney-Metabolic Syndrome” emphasizing that cardiovascular and metabolic health are inextricable [36]. In the context of EVT, reperfusion-related stress and procedural blood pressure variability may further amplify these pathological processes in patients with high CTI [37].

An important question is whether CTI predicts outcomes independently or primarily through subsequent cardiac injury (stroke–heart syndrome). Mediation analysis (Table 5) reveals CTI operates through both pathways: 36.4% of the effect is mediated through dynamic troponin elevation, while 63.6% represents direct effects independent of cardiac injury (both *p* < 0.01). The indirect pathway reflects increased vulnerability to stroke-induced cardiac injury, while the direct effect likely captures pre-existing inflammatory-metabolic dysregulation that independently impairs stroke recovery. Clinically, CTI’s early availability and substantial direct effect (63.6%) indicate it provides prognostic information complementary to troponin monitoring.

An unexpected finding of this study was that the proportion of patients achieving complete functional independence (mRS 0) in the Q1 group with the lowest CTI was lower than that in the Q2 group, although the Q1 group represented the lowest inflammation-metabolic burden. Several factors may explain the unexpectedly low proportion of mRS 0 in group Q1. Firstly, low TG levels may reflect malnutrition, sarcopenia or chronic wasting diseases—these conditions are associated with reduced physiological reserves and impaired neural plasticity. This is consistent with the “obesity paradox” observed in cardiovascular diseases, where extremely low values of metabolic indicators are actually associated with poorer outcomes in some populations [38]. Secondly, achieving mRS 0 requires going beyond the factors of inflammation-metabolic control. Full functional independence requires the restoration of subtle cognitive, motor and psychosocial functions, which may depend on rehabilitation accessibility and social support—factors that are not captured by CTI. Importantly, this model does not diminish the clinical practicality of CTI in risk stratification. The main value of CTI lies in identifying patients at high risk of adverse outcomes (mRS 3–6 and death) that require close monitoring and intervention.

Although we adopted some methods, such as comprehensive determination of outcome indicators (no loss to follow-up); comprehensive covariate adjustment (18 covariates in Model 3); multiple sensitivity analyses and mediation analyses; and consistent results across subgroups (no significant interaction), we still need to acknowledge the inherent limitations of the retrospective design itself. In addition, this study has several other limitations. Firstly, our research focused on patients with LVO in the anterior circulation who received EVT—this was a design choice aimed at studying this high-risk group. Although this focus facilitated a rigorous assessment of the mechanisms of cardiac injury, it limited the direct generalization to patients with mild stroke or those not suitable for EVT. It is necessary to validate in a broader stroke population, including non-LVO stroke patients and those who only received thrombolysis or best medical treatment, to evaluate the predictive value of CTI across the full spectrum of stroke severity and treatment modalities. Secondly, a significant proportion of patients (361/873) were excluded due to missing paired troponin measurements. During the study period, our hospital utilized separate electronic health record (EHR) systems for the emergency department (ED) and inpatient wards. This system incompatibility particularly affected patients admitted during 2020–2022, before full EHR integration was achieved. Importantly, this data loss was due to technical factors unrelated to patient clinical characteristics, as evidenced by the lack of significant differences between included and excluded patients (Table S8). Thirdly, we lack comprehensive baseline cardiac assessment data, including left ventricular ejection fraction (LVEF), detailed cardiac structural disease evaluation, and levels of natriuretic peptides (BNP/NT-proBNP). Although we adjusted for the history of CAD and AF, the lack of quantitative cardiac function parameters limits our ability to fully distinguish between pre-existing cardiac dysfunction and injury caused by acute stroke. Future studies that incorporate systematic cardiac ultrasound examinations and cardiac biomarkers will be able to describe the baseline cardiac status in greater detail. Finally, we acknowledge that CTI, measured in the acute phase, may reflect both metabolic status and acute stress responses. Although we adjusted for multiple severity markers and used strict fasting protocols, we cannot completely rule out acute stress contributions—a fundamental interpretative challenge for any biomarker measured in the acute phase [28]. The absence of HbA1c, pre-stroke metabolic profiles, or acute stress biomarkers (e.g., cortisol) limits our ability to fully quantify the relative contributions of chronic versus acute components. Future prospective studies incorporating pre-stroke HbA1c, and serial CTI measurements would help definitively establish the temporal nature of CTI components and strengthen causal inference.

## 5. Conclusions

In this two-center retrospective cohort study of anterior-circulation LVO patients undergoing EVT, we found that elevated admission CTI was associated with both increased risk of SHS (characterized by dynamic cTnI elevation) and worse 90-day functional outcomes and mortality.

## Figures and Tables

**Table 1:** Patient demographics and baseline characteristics.

Characteristic	CTI Group				*p*-Value
	Q1 [6.47,8.69) *n* = 123	Q2 [8.69,9.32) *n* = 123	Q3 [9.32,10) *n* = 123	Q4 [10,12.6] *n* = 124	
Age, Median (Q1, Q3)	68 (58, 76)	70 (60, 76)	71 (61, 78)	69 (60, 77)	0.610
Female, n (%)	46 (37.4%)	44 (35.8%)	50 (40.7%)	67 (54.5%)	0.012
Hypertension, n (%)	74 (60.2%)	86 (69.9%)	87 (70.7%)	83 (66.9%)	0.279
Diabetes mellitus, n (%)	19 (15.4%)	29 (23.6%)	37 (30.1%)	56 (45.2%)	<0.001
Hyperlipidemia, n (%)	17 (13.8%)	26 (21.1%)	36 (29.3%)	46 (37.1%)	<0.001
History of coronary artery disease, n (%)	33 (26.8%)	26 (21.1%)	28 (22.8%)	33 (26.6%)	0.657
History of atrial fibrillation, n (%)	48 (39.0%)	51 (41.5%)	44 (35.8%)	48 (38.7%)	0.839
Smoking status, n (%)	36 (29.3%)	29 (23.6%)	34 (27.6%)	25 (20.2%)	0.346
Prior stroke, n (%)	24 (19.5%)	18 (14.6%)	31 (25.2%)	18 (14.5%)	0.099
Baseline NIHSS, Median (Q1, Q3)	16 (11, 26)	15 (10, 25)	18 (10, 25)	18 (11, 25)	0.499
Baseline ASPECTS, Median (Q1, Q3)	8.00 (6.00, 9.00)	8.00 (6.00, 9.00)	8.00 (5.00, 9.00)	8.00 (5.00, 9.00)	0.657
TOAST subtype, n (%)					0.001
cardioembolism	50 (40.7%)	61 (49.6%)	42 (34.1%)	54 (43.5%)	
large artery atherosclerosis	54 (43.9%)	57 (46.3%)	64 (52.0%)	66 (53.2%)	
other or undetermined	19 (15.4%)	5 (4.1%)	17 (13.8%)	4 (3.2%)	
Occlusion site, n (%)					0.624
ICA	59 (48.0%)	67 (54.5%)	73 (59.3%)	67 (54.0%)	
M1	47 (38.2%)	40 (32.5%)	39 (31.7%)	45 (36.3%)	
M2	17 (13.8%)	16 (13.0%)	11 (8.9%)	12 (9.7%)	
Final mTICI, n (%)					0.289
0–2a	5 (4.1%)	9 (7.3%)	10 (8.1%)	13 (10.5%)	
2b–3	118 (95.9%)	114 (92.7%)	113 (91.9%)	111 (89.5%)	
sICH, n (%)	9 (7.3%)	15 (12.2%)	8 (6.5%)	13 (10.5%)	0.365
Malignant cerebral edema (post-EVT), n (%)	23 (18.7%)	29 (23.6%)	26 (21.1%)	32 (25.8%)	0.570
TG, mg/dL, Median (Q1, Q3)	66 (49, 87)	97 (70, 127)	129 (84, 167)	181 (112, 287)	<0.001
CRP, mg/L, Median (Q1, Q3)	1 (0, 1)	2 (1, 4)	4 (1, 10)	15 (3, 34)	<0.001
FBG, mg/dL, Median (Q1, Q3)	6.4 (5.6, 7.4)	7.0 (6.0, 8.1)	7.5 (6.5, 9.5)	9.5 (7.6, 13.9)	<0.001
cTnI trajectory, n (%)					<0.001
1	85 (69.1%)	80 (65.0%)	63 (51.2%)	51 (41.1%)	
2	8 (6.5%)	10 (8.1%)	16 (13.0%)	24 (19.4%)	
3	30 (24.4%)	33 (26.8%)	44 (35.8%)	49 (39.5%)	
eGFR, mL/min/1.73 m^2^, Median (Q1, Q3)	78.5 (58.3, 92.1)	76.2 (56.8, 90.5)	72.4 (54.1, 88.7)	70.8 (51.2, 87.3)	0.127

Abbreviations: NIHSS, National Institutes of Health Stroke Scale; ASPECTS, Alberta Stroke Program Early CT Score; TOAST, Trial of Org 10172 in Acute Stroke Treatment; ICA, internal carotid artery; M1/M2, middle cerebral artery segments; mTICI, modified Thrombolysis in Cerebral Infarction; sICH, symptomatic intracranial hemorrhage; CRP, C-reactive protein; FBG, fasting blood glucose; TG, triglycerides; cTnI, cardiac troponin I; eGFR, estimated glomerular filtration rate.

**Table 2:** Association between CTI and trajectory of cTnI change.

Characteristic	Model 1			Model 2			Model 3		
	OR	95% CI	*p*-Value	OR	95% CI	*p*-Value	OR	95% CI	*p*-Value
CTI (continuous)	1.55	1.29, 1.86	<0.001	1.56	1.27, 1.92	<0.001	1.56	1.26, 1.94	<0.001
CTI									
Q1 [6.47,8.69)	Ref	Ref		Ref	Ref		Ref	Ref	
Q2 [8.69,9.32)	1.20	0.71, 2.05	0.498	1.20	0.68, 2.13	0.533	1.21	0.66, 2.21	0.537
Q3 [9.32,10)	2.13	1.27, 3.59	0.004	2.21	1.26, 3.90	0.006	2.33	1.29, 4.22	0.005
Q4 [10,12.6]	3.20	1.90, 5.41	<0.001	3.44	1.90, 6.20	<0.001	3.49	1.87, 6.51	<0.001
*p* for trend			<0.001			<0.001			<0.001

Abbreviations: CI = Confidence Interval, OR = Odds Ratio; Model 1: no covariates were adjusted. Model 2: adjusted for sex, age, hypertension, diabetes mellitus, hyperlipidemia, coronary artery disease, history of atrial fibrillation, smoking status, and prior stroke. Model 3: additionally adjusted for occlusion site (ICA/M1/M2), TOAST subtype (cardioembolism/large artery atherosclerosis/other or undetermined), baseline NIHSS, baseline ASPECTS, first-line thrombectomy technique (no device pass/aspiration/stent retriever/combined), final reperfusion status (mTICI 2b–3 vs. 0–2a), sICH, and malignant cerebral edema.

**Table 3:** Association between CTI and poor functional outcome and 90-day mortality.

	Poor Functional Outcome (mRS 3–6)					
	CTI (Continuous)	Q1 [6.47,8.69)	Q2 [8.69,9.32)	Q3 [9.32,10)	Q4 [10,12.6]	*p* for Trend
Model 1	1.33 (1.11, 1.59) *p* = 0.002	Ref	0.82 (0.50, 1.36) *p* = 0.444	1.56 (0.93, 2.60) *p* = 0.092	2.13 (1.25, 3.60) *p* = 0.005	<0.001
Model 2	1.45 (1.18, 1.77) *p* < 0.001	Ref	0.88 (0.51, 1.50) *p* = 0.626	1.63 (0.94, 2.85) *p* = 0.083	2.89 (1.59, 5.26) *p* < 0.001	0.011
Model 3	1.47 (1.16, 1.86) *p* = 0.001	Ref	0.89 (0.48, 1.64) *p* = 0.704	1.74 (0.92, 3.29) *p* = 0.089	3.04 (1.53, 6.05) *p* = 0.001	<0.001
	90d mortality					
Model 1	1.52 (1.23, 1.87) *p* < 0.001	Ref	1.18 (0.61, 2.29) *p* = 0.615	1.74 (0.93, 3.25) *p* = 0.086	2.45 (1.33, 4.51) *p* = 0.004	0.001
Model 2	1.64 (1.30, 2.08) *p* < 0.001	Ref	1.37 (0.67, 2.79) *p* = 0.383	1.99 (1.00, 3.95) *p* = 0.050	3.11 (1.55, 6.24) *p* = 0.001	<0.001
Model 3	1.62 (1.25, 2.11) *p* < 0.001	Ref	1.20 (0.56, 2.59) *p* = 0.642	1.88 (0.89, 3.94) *p* = 0.096	2.82 (1.33, 6.00) *p* = 0.007	0.003

Abbreviations: CI = Confidence Interval, OR = Odds Ratio. Model 1: no covariates were adjusted. Model 2: adjusted for sex, age, hypertension, diabetes mellitus, hyperlipidemia, coronary artery disease, history of atrial fibrillation, smoking status, and prior stroke. Model 3: additionally adjusted for occlusion site (ICA/M1/M2), TOAST subtype (cardioembolism/large artery atherosclerosis/other or undetermined), baseline NIHSS, baseline ASPECTS, first-line thrombectomy technique (no device pass/aspiration/stent retriever/combined), final reperfusion status (mTICI 2b–3 vs. 0–2a), sICH, and malignant cerebral edema.

**Table 4:** Association between cTnI trajectory and 90-day clinical outcomes.

Outcome	Group 1 (No Myocardial Injury)	Group 2 (Non-Dynamic Elevation)		Group 3 (Dynamic Elevation)	
		aOR (95% CI)	*p*	aOR (95% CI)	*p*
90-day Poor Functional Outcome	Ref	1.39 (0.70, 2.77)	0.461	8.73 (4.53, 16.83)	<0.001
90-day All-cause Mortality	Ref	1.67 (0.69, 4.03)	0.250	6.12 (3.40, 11.19)	<0.001

Adjusted for sex, age, hypertension, diabetes mellitus, hyperlipidemia, coronary artery disease, history of atrial fibrillation, smoking status, and prior stroke, occlusion site (ICA/M1/M2), TOAST subtype (cardioembolism/large artery atherosclerosis/other or undetermined), baseline NIHSS, baseline ASPECTS, first-line thrombectomy technique (no device pass/aspiration/stent retriever/combined), final reperfusion status (mTICI 2b–3 vs. 0–2a), sICH, and malignant cerebral edema.

**Table 5:** Mediation analysis: direct and indirect effects of CTI on 90-day poor functional outcome.

Effect Component	Coefficient	95% CI	*p*
Total Effect	0.385	0.218–0.562	<0.001
Indirect Effect (ACME)	0.140	0.067–0.232	<0.001
Direct Effect (ADE)	0.241	0.104–0.395	0.002
Proportion Mediated	36.4%	21.5–57.5%	<0.001

ACME = Average Causal Mediation Effect (indirect effect through dynamic troponin elevation); ADE = Average Direct Effect (effect not mediated by troponin). Adjusted for all covariates in Table 3 Model 3.

## Data Availability

The datasets used and/or analyzed during the current study are available from the corresponding author on reasonable request.

## Supplementary Materials

The following supporting information can be downloaded at: https://www.mdpi.com/article/10.3390/jcdd13050179/s1, Table S1. Timeline of key clinical events and blood sample collection; Table S2. Association between CTI quartiles and clinical outcomes; Table S3. Incremental discriminative value; Table S4. Subgroup analysis for poor functional outcome (mRS 3–6); Table S5. Subgroup analysis for 90-day mortality; Table S6. Association between CTI and troponin trajectory, stratified by renal function; Table S7. The sensitivity analysis on poor functional outcome (mRS 3–6); Table S8. Baseline characteristics of included patients and patients excluded due to missing paired troponin measurements.

## Abbreviations

The following abbreviations are used in this manuscript:

AF	Atrial Fibrillation
AIS	Acute Ischemic Stroke
aOR	Adjusted Odds Ratio
ASPECTS	Alberta Stroke Program Early CT Score
CI	Confidence Interval
cTnI	Cardiac troponin I
CKM	Cardiovascular-Kidney-Metabolic
CRP	C-reactive Protein
CTI	C-reactive Protein–Triglyceride–Glucose Index
DSA	Digital Subtraction Angiography
EVT	Endovascular Thrombectomy
FBG	Fasting Blood Glucose
ICA	Internal Carotid Artery
IQR	Interquartile Range
LVO	Large-Vessel Occlusion
MCA	Middle Cerebral Artery
MCE	Malignant Cerebral Edema
mRS	Modified Rankin Scale
mTICI	Modified Thrombolysis in Cerebral Infarction
NIHSS	National Institutes of Health Stroke Scale
RCS	Restricted Cubic Spline
SHS	Stroke–Heart Syndrome
