# Supplementary material for: Association of the C-Reactive Protein–Triglyceride–Glucose Index with Stroke–Heart Syndrome and Clinical Prognosis in Patients Undergoing Endovascular Treatment
Source: J Cardiovasc Dev Dis. 2026 Apr 25;13(5):179. doi: 10.3390/jcdd13050179 (PMC13207347; doi:10.3390/jcdd13050179)
Supplement: Supplementary file 1 [file jcdd-13-00179-s001.zip › jcdd-4141854-supplementary.pdf]

Supplements：

Table S1. Timeline of Key Clinical Events and Blood Sample Collection

Time Interval	Median (IQR)
Symptom onset to hospital admission	2.8 hours (1.5–4.7)
Symptom onset to baseline blood sampling (CRP, cTnI)	3.2 hours (1.7–5.2)
Admission to EVT initiation (groin puncture)	1.5 hours (1.2–2.1)
Admission to fasting blood sampling (TG, FBG)	17 hours (13–23)

Table S2. Association between CTI quartiles and clinical outcomes

Characteristic	CTI group				p-value
	Q1 [6.47,8.69) N = 123	Q2 [8.69,9.32) N = 123	Q3 [9.32,10) N = 123	Q4 [10,12.6] N = 124	
Poor functional outcome, n (%)	67 (54.5%)	61 (49.6%)	80 (65.0%)	89 (71.8%)	0.001
All cause death at 90 days, n (%)	20 (16.3%)	23 (18.7%)	31 (25.2%)	40 (32.3%)	0.013

Table S3. Incremental Discriminative Value

Outcome	Model	AUC (95% CI)	ΔAUC vs Base	Z	DeLong P	vs CRP Model
90-Day Poor Functional Outcome						
	Base Model	0.807 (0.769-0.845)	-	-	-	-
	Base + CRP	0.821 (0.785-0.858)	+0.014	1.89	0.059	-
	Base + CTI	0.826 (0.790-0.862)	+0.019	2.24	0.025	ΔAUC = +0.005 P = 0.497 LRT: χ^2=4.82, P=0.028
90-Day All-Cause Mortality						
	Base Model	0.819 (0.778-0.860)	-	-	-	-
	Base +	0.824	+0.005	0.72	0.472	-

Outcome	Model	AUC (95% CI)	ΔAUC vs Base	Z	DeLong P	vs CRP Model
	CRP	(0.782-0.865)				
	Base + CTI	0.832 (0.792-0.872)	+0.013	2.08	0.038	ΔAUC = +0.008 P = 0.263 LRT: χ^2=5.90, P=0.015

P values from DeLong's test for correlated ROC curves. LRT, Likelihood ratio test comparing Base+CTI vs Base+CRP (df=1). AUC, area under the curve; CRP, C-reactive protein; CTI, C-reactive protein(CRP) -triglyceride(TG)–glucose index

Table S4. Subgroup Analysis for poor functional outcome (mRS 3-6)

Subgroup	N	OR (95% CI)	P value	P for interaction
Overall	493	1.33 (1.11-1.59)	<0.001	
Sex				0.666
Male	285	1.28 (1.01-1.63)	0.040	
Female	207	1.39 (1.05-1.85)	0.023	
Hypertension				0.587
NO	163	1.14 (0.85-1.54)	0.377	
YES	330	1.44 (1.15-1.82)	0.002	
Diabetes				0.789
NO	352	1.32 (1.05-1.64)	0.015	
YES	141	1.42 (1.02-2.01)	0.040	
Hyperlipidemia				0.209
NO	368	1.28 (1.03-1.54)	0.023	
YES	125	1.70 (1.14-2.52)	0.009	
Coronary artery disease				0.866
NO	373	1.34 (1.09-1.65)	0.006	
YES	120	1.29 (0.88-1.88)	0.183	
History of atrial fibrillation				0.992
NO	302	1.34 (1.07-1.67)	0.001	
YES	191	1.34 (0.97-1.84)	0.072	
Prior stroke				0.890
NO	402	1.33 (1.10-1.62)	0.004	
YES	91	1.39 (0.84-2.28)	0.199	

Table S5. Subgroup Analysis for 90-day mortality

Subgroup	N	OR (95% CI)	P value	P for interaction

Subgroup	N	OR (95% CI)	P value	P for interaction
Overall	493	1.52 (1.23-1.87)	<0.001	
Sex				0.141
Male	285	1.74 (1.27-2.38)	0.001	
Female	207	1.26 (0.95-1.68)	0.113	
Hypertension				0.248
NO	163	1.27 (0.89-1.82)	0.190	
YES	330	1.65 (1.27-2.14)	<0.001	
Diabetes				0.364
NO	352	1.68 (1.29-2.19)	<0.001	
YES	141	1.35 (0.91-2.00)	0.136	
Hyperlipidemia				0.523
NO	368	1.52 (1.20-1.93)	0.001	
YES	125	1.81 (1.11-2.95)	0.016	
Coronary artery disease				0.949
NO	373	1.51 (1.17-1.97)	0.002	
YES	120	1.54 (1.07-2.21)	0.021	
History of atrial fibrillation				0.072
NO	302	1.82 (1.37-2.42)	<0.001	
YES	191	1.23 (0.89-1.69)	0.212	
Prior stroke				0.802
NO	402	1.50 (1.20-1.88)	<0.001	
YES	91	1.62 (0.92-2.84)	0.092	

Table S6. Association between CTI and Troponin Trajectory, Stratified by Renal Function

Subgroup	CTI (continuous), OR (95% CI)*	p-value	P for interaction
eGFR ≥60 mL/min/1.73m² (n=362)	1.62 (1.24–2.12)	<0.001	0.614
eGFR <60 mL/min/1.73m² (n=131)	1.48 (0.96–2.28)	0.076	

*OR from ordinal logistic regression represents the odds of being in a higher troponin trajectory category (worse cardiac injury) per 1-unit increase in CTI.
Adjusted for: age, sex, hypertension, diabetes mellitus, hyperlipidemia, coronary artery disease, atrial fibrillation, smoking status, prior stroke, occlusion site, TOAST subtype, baseline NIHSS, baseline ASPECTS, first-line thrombectomy technique, final reperfusion status (mTICI 2b–3 vs 0–2a), sICH, and malignant cerebral edema.

Table S7. The sensitivity analysis on poor functional outcome (mRS 3-6)

Subgroup	CTI Levels	OR (95% CI)	P	P for interaction
Troponin trajectories				0.371

Subgroup	CTI Levels	OR (95% CI)	P	P for interaction
No myocardial injury	Q1 [6.47,8.69)	Ref		
	Q2 [8.69,9.32)	0.87 (0.33-1.55)	0.663	
	Q3 [9.32,10)	2.29 (1.00-5.27)	0.050	
	Q4 [10,12.6]	3.94 (1.46-6.71)	0.018	
Non-dynamic elevation	Q1 [6.47,8.69)	Ref		
	Q2 [8.69,9.32)	1.54 (0.25-36.32)	0.388	
	Q3 [9.32,10)	2.18 (0.27-18.66)	0.486	
	Q4 [10,12.6]	4.26 (1.08-16.85)	0.039	
Dynamic elevation	Q1 [6.47,8.69)	Ref		
	Q2 [8.69,9.32)	0.83 (0.31-2.24)	0.718	
	Q3 [9.32,10)	1.00 (0.80-2.53)	0.998	
	Q4 [10,12.6]	2.04 (1.42-2.58)	0.003	
Adjusted for sex, age, hypertension, diabetes mellitus, hyperlipidemia, coronary artery disease, history of atrial fibrillation, smoking status, and prior stroke, occlusion site (ICA/M1/M2), TOAST subtype (cardioembolism/large artery atherosclerosis/other or undetermined), baseline NIHSS, baseline ASPECTS, first-line thrombectomy technique (no device pass/aspiration/stent retriever/combined), final reperfusion status (mTICI 2b–3 vs 0–2a), sICH, and malignant cerebral edema.				

Table S8. Baseline Characteristics of Included Patients and Patients Excluded Due to Missing Paired Troponin Measurements

Characteristic	Included (N=493)	Excluded (N=361)	p-value
Demographics			
Age, years, Median (Q1, Q3)	69 (60, 77)	70 (61, 77)	0.521
Female, n (%)	207 (42.0)	154 (42.7)	0.845
Vascular Risk Factors			
Hypertension, n (%)	330 (66.9)	245 (67.9)	0.753
Diabetes mellitus, n (%)	141 (28.6)	109 (30.2)	0.612
Hyperlipidemia, n (%)	125 (25.4)	87 (24.1)	0.664
History of coronary artery disease, n (%)	120 (24.3)	84 (23.3)	0.721
History of atrial fibrillation, n (%)	191 (38.7)	138 (38.2)	0.881
Smoking status, n (%)	124 (25.2)	93 (25.8)	0.847
Prior stroke, n (%)	91 (18.5)	72 (19.9)	0.591
Baseline Clinical Severity			
Baseline NIHSS, Median (Q1, Q3)	17 (11, 25)	17 (11, 24)	0.782
Baseline ASPECTS, Median (Q1, Q3)	8 (6, 9)	8 (6, 9)	0.891

Characteristic	Included (N=493)	Excluded (N=361)	p-value
TOAST subtype, n (%)			0.756
Cardioembolism	207 (42.0)	157 (43.5)	
Large artery atherosclerosis	241 (48.9)	171 (47.4)	
Other or undetermined	45 (9.1)	33 (9.1)	
Occlusion site, n (%)			0.918
ICA	266 (54.0)	197 (54.6)	
M1	171 (34.7)	123 (34.1)	
M2	56 (11.4)	41 (11.4)	
Procedural Outcomes			
Final mTICI 2b-3, n (%)	456 (92.5)	331 (91.7)	0.658
sICH, n (%)	45 (9.1)	36 (10.0)	0.677
Malignant cerebral edema, n (%)	110 (22.3)	84 (23.3)	0.738

Abbreviations: NIHSS, National Institutes of Health Stroke Scale; ASPECTS, Alberta Stroke Program Early CT Score; TOAST, Trial of Org 10172 in Acute Stroke Treatment; ICA, internal carotid artery; M1/M2, middle cerebral artery segments; mTICI, modified Thrombolysis in Cerebral Infarction; sICH, symptomatic intracranial hemorrhage; CRP, C-reactive protein; FBG, fasting blood glucose; TG, triglycerides; cTnI, cardiac troponin I.
